# A CSF disease-associated macrophage signature defines progressive multiple sclerosis

**DOI:** 10.1186/s12974-026-03861-9

**Published:** 2026-05-13

**Authors:** Anna-Lena Börsch, Frederike Riethues, Andreas Schulte-Mecklenbeck, Xuesong Wang, Michael Heming, I-Na Lu, Louisa Müller-Miny, Heinz Wiendl, Catharina C. Gross, Raphaël Bernard-Valnet, Gerd Meyer zu Hörste

**Affiliations:** 1 Department of Neurology, Medical Faculty, University Hospital Münster, Münster, Germany; 2 Department of Neurology and Neurophysiology, University Hospital Freiburg, Freiburg, Germany; 3 Neurology Service, Department of Clinical Neurosciences, Lausanne University Hospital Centre Hospitalier Universitaire Vaudois (CHUV) and Lausanne University (UNIL), Lausanne, Switzerland

**Keywords:** Single-cell RNA-seq, Cerebrospinal fluid, Progressive multiple sclerosis

## Abstract

**Objective:**

Progression in multiple sclerosis (MS) often corresponds to irreversible disability in MS patients. Cellular changes in the cerebrospinal fluid (CSF) have provided biomarkers and mechanisms in relapsing-remitting MS (RRMS) but remain understudied in primary and secondary progressive MS (summarized herein as PMS).

**Methods:**

We combined retrospective flow cytometry of CSF cells from RRMS (*n* = 169), PMS (*n* = 56), and non-inflammatory controls (*n* = 74) with prospective CSF single-cell transcriptomics of 35 individuals (11 controls, 12 RRMS, and 12 PMS) and with confirmatory CSF ELISA. Available CSF single-cell data from age-matched and Alzheimer’s disease (AD) patients served as additional controls.

**Results:**

Proportions of CD14^+^ monocytes in CSF are increased in PMS and correlated with clinical surrogate markers of progression. Transcriptionally, these monocytes resembled border-associated macrophages (BAM)-like cells with a chronically activated antigen-presenting phenotype. Additionally, these monocytes shared some features with disease-associated microglia/macrophages (DAM), previously identified in neurodegeneration. Induction of DAM-associated molecules, including transcribed and soluble TREM2 (sTREM2), characterized secondary progressive MS (SPMS) and supported its differential diagnosis.

**Interpretation:**

We thus identified MS stage-specific CSF signatures and shared cellular features of degeneration detectable in CSF of PMS patients.

**Graphical Abstract:**

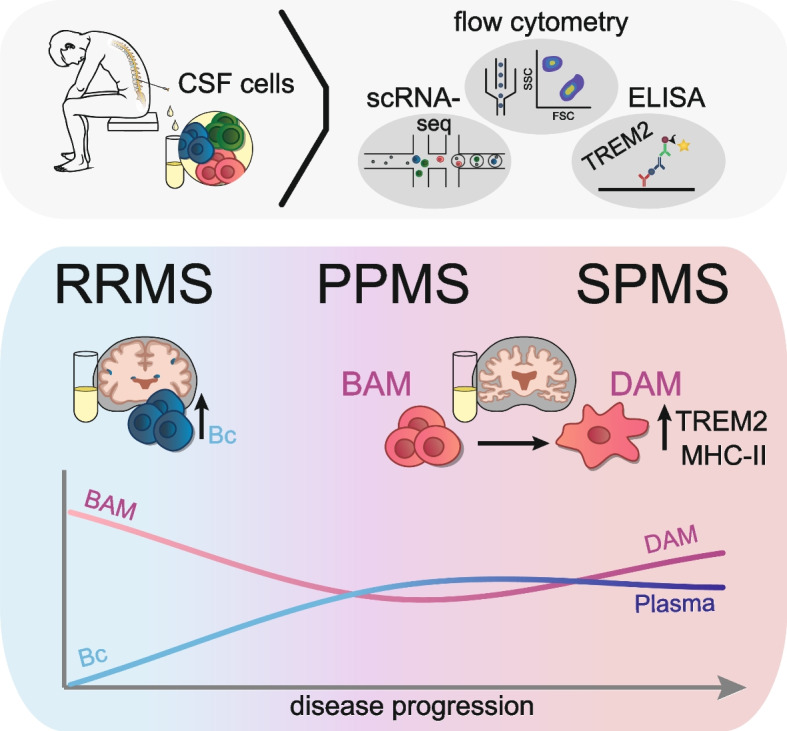

**Supplementary Information:**

The online version contains supplementary material available at 10.1186/s12974-026-03861-9.

## Introduction

Multiple sclerosis (MS) affects nearly 3 million people worldwide and follows different clinical courses. At onset, ~ 90% of patients present with relapsing-remitting MS (RRMS), but 60-90% transition to secondary progressive MS (SPMS) within two decades [1]. Modern disease-modifying therapies have significantly reduced and delayed this conversion [2]. In addition, ~ 15% of patients develop progressive disability from disease onset without relapses and are then classified as primary progressive MS (PPMS).

MS is best conceptualized as a continuum between inflammation and neurodegeneration [3]. In RRMS, inflammation dominates, while progressive MS (PMS) is driven increasingly by neurodegeneration, axonal loss, and reduced reparative capacity. Aging amplifies this shift through processes such as “inflammaging,” diminished neural plasticity, and greater vulnerability to degeneration [4]. Crucially, progression can already manifest during the RRMS phase, independent of relapses — a phenomenon termed “progression independent of relapse activity” (PIRA) [5].

Pathological studies point to axonal injury and compartmentalized inflammation within the central nervous system (CNS) as central drivers of progression. Smoldering white-matter lesions and leptomeningeal inflammation are now recognized as key sites of such immune activity [6]. Imaging markers, such as paramagnetic rim lesions and cortical gray-matter lesions, capture aspects of these processes and have been repeatedly linked to accelerated brain atrophy and disability progression [7, 8].

The mechanisms driving RRMS are well characterized, but the cellular immune mechanisms driving PMS remain largely elusive. Because PMS patients rarely undergo lumbar puncture in the absence of relapses, cerebrospinal fluid (CSF) is less available for research — particularly for cellular analyses that require prespecified processing conditions [9]. Current knowledge, therefore, stems largely from two sources: CSF proteomic studies showing that aging reshapes immune signatures toward age-related rather than MS-specific profiles [10] and autopsy studies that highlight the role of myeloid cells, particularly disease-associated microglia (DAM) and macrophages, in potentially driving progression [11, 12]. These insights align with the recent success of Bruton’s tyrosine kinase (BTK) inhibitors, the first therapy to demonstrate efficacy in non-relapsing SPMS acting on myeloid cells as well as on B cells [13, 14].

CSF offers a unique mechanistic and diagnostic window into CNS disease mechanisms. Produced by the choroid plexus and circulating throughout the CNS, it can reflect both inflammatory and neurodegenerative processes. High-dimensional flow cytometry and single-cell transcriptomics have already mapped complex immune alterations in RRMS [15]. Building on this work, we here combined high-dimensional CSF flow cytometry in a large cohort of patients with MS with single-cell analyses of CSF leukocytes from 35 patients, aiming to delineate the immune changes associated with MS progression. Myeloid cells showed a relative increase in the CSF in PMS and exhibited DAM-like features with signs of increased antigen-presenting capacity. Mechanisms of progression may thus be partially shared between chronic MS and primary neurodegenerative diseases.

## Methods

### Study cohort and analysis of the flow cytometry cohort

**Fig. 1:**
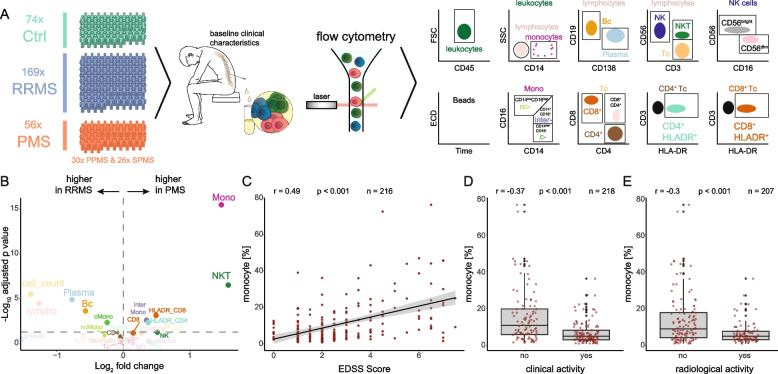
Flow cytometry analysis of the cerebrospinal fluid demonstrates a relative increase of monocytes in progressive multiple sclerosis. **A** Schematic of the study design. Cerebrospinal fluid (CSF) of 74 non-inflammatory control subjects (Ctrl), 169 relapsing–remitting multiple sclerosis (RRMS) patients, and 56 patients with progressive multiple sclerosis (PMS) was studied with a standardized flow cytometry panel for different immune cells and CSF routine parameters. **B** Flow cytometry data of total cell count, glucose ratio, lactate (mmol/l), B cells, classical monocytes (cMono), intermediate monocytes (interMono), non-classical monocytes (ncMono), CD4^+^ T cells, CD4^+^CD8^+^ cells, HLADR^+^CD4^+^ cells (HLADR_CD4), CD8^+^ T cells, HLADR^+^CD8^+^ cells (HLADR_CD8), T cells, monocytes (mono), natural killer cells (NK), natural killer T cells (NKT), plasma cells (plasma), CD56^dim^ NK (dim), CD56^bright^ NK (bright). Volcano plots depicting changes in parameter/cell type abundance in RRMS vs. PMS. Logarithmic two-fold change (Log2FC) of cell type abundance is plotted against negative logarithmic adjusted *p*-value for multiple hypotheses (pvalue_adj). The horizontal line visualizes the significance threshold (adjusted *p*-value 0.05). **C**-**E** Correlation analysis between the percentage of monocytes and (**C**) expanded disability status scale (EDSS score), (**D**) clinical activity within the last 4 weeks, and (**E**) radiological activity within the last 4 weeks. For (**C**), data are shown as correlation plots with a linear regression line (solid line) and with its confidence interval in grey, and in (**D**) and (**E**), data are represented in dot plots. The Pearson correlation coefficient (R), the *p*-value (p), and the number of included patients (n) are depicted above the respective plots

All CSF samples collected for diagnostic purposes were analyzed using a standardized flow cytometry panel at our center (Methods, Fig. 1 A), thus providing a large dataset for hypothesis generation. By querying electronic health records of the last 10 years, we collected CSF flow cytometry data of 74 non-inflammatory controls (Ctrl; 46 females, 28 males), 169 relapsing–remitting multiple sclerosis (RRMS) patients (124 females, 45 males), and 56 progressive MS (PMS) patients (33 females, 23 males) (Suppl. Tab. 1, Suppl. Figure 1 A), which is partly published in Gross et al. [16] In short, non-inflammatory Ctrl subjects were patients with somatoform disorders or those who required lumbar puncture during surgical procedures. All RRMS patients had not been on immunomodulatory drugs prior to CSF analysis and were diagnosed according to the 2017 revised McDonald criteria [17]. PMS, summarizing primary and secondary progressive MS here, was defined as clinical deterioration or MRI activity without relapses [18]. The research adhered to the principles outlined in the Declaration of Helsinki and gained approval from the local ethical authorities (2010—262—f-S, 2011—665—f-S, 2013—350—f-S, 2014—068—f-S and 2016—053—f-S). Written informed consent was obtained from all participating patients.

The flow cytometry data were processed and generated as described in Gross et al. [16]. Briefly, CSF cells were stained in 100 µl FC buffer supplemented with fluorochrome-conjugated antibodies directed against CD3 (UCHT1), CD4 (13B8.2), CD8 (B9.11), CD14 (RM052), CD16 (3G8), CD19 (J3-119), CD45 (J33), CD56 (N901), CD138 (BA38), and HLA-DR (Immu-357) (all Beckman Coulter, clones in brackets). Samples were acquired using a Navios flow cytometer (Beckman Coulter), and results were analyzed using Kaluza 2.1 software (Beckman Coulter). To summarize, SSC^int^CD14^+^ monocytes (subdivided into CD14^high^CD16^−^, CD14^+^CD16^+^, CD14^low^CD16^high^), CD19^high^CD138^−^ B cells, CD19^low^CD138^high^ plasma cells, CD56^+^CD3^−^ NK cells, CD56^+^CD3^+^ NKT, CD3^+^CD56^−^ T cells, and HLA-DR^+^ CD4/CD8^+^ T cells were analyzed by flow cytometry according to the gating strategy of Gross et al. (Suppl. Figure 7 in [16] and Fig. 1 A). Routine CSF parameters (CSF cell count, lymphocytes, granulocytes, erythrocytes, other cells, IgG/A/M ratios, protein, glucose, lactate, albumin, and oligoclonal bands (OCBs)) were analyzed in a standardized operating procedure, as previously described [16, 19, 20]. Briefly, 50 µl CSF cells were stained with methyl violet (10 µl), classified microscopically into erythrocytes, lymphocytes, granulocytes, and other cells, and furthermore counted in a Fuchs-Rosenthal chamber. Total protein, albumin, and immunoglobulins were measured by nephelometry (BN ProSpec, Siemens), and glucose and lactate were determined in cell-free CSF using a SuperGL compact analyzer (Hitado). Oligoclonal bands were assessed by isoelectric focusing with silver nitrate staining.

Group comparisons were performed using the R package *rstatix v0.7.2*. For categorical variables, pairwise Fisher’s exact tests were applied. For continuous variables, Wilcoxon rank-sum tests were used for two-group comparisons and Dunn’s tests for multiple-group comparisons, with Benjamini–Hochberg correction for multiple testing. Cell-type proportion differences were analyzed with the propeller.ttest function from the *speckle v1.8.0* package using robust variance estimation, including age and sex as covariates where indicated. Correlations between continuous parameters (e.g., monocyte frequency and radiological, clinical, or disability measures) were assessed using Pearson’s correlation.

### Study cohort and acquisition of study material for scRNA-seq

**Fig. 2:**
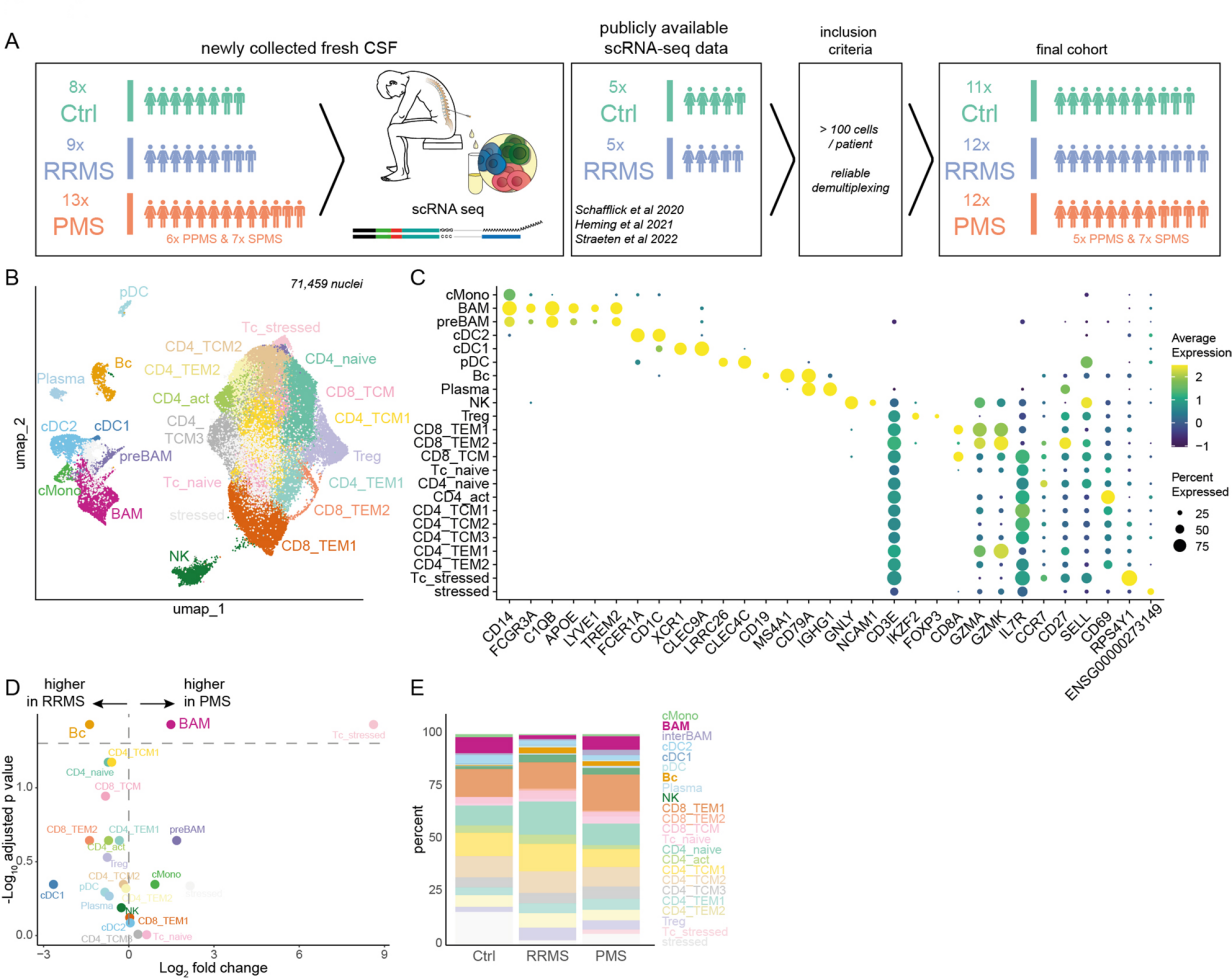
Single-cell transcriptomic profiling of the cerebrospinal fluid in progressive multiple sclerosis highlights myeloid rather than lymphocyte involvement. **A** Schematic of the study design illustrating the source of the cerebrospinal fluid (CSF) single-cell data (newly collected and publicly available 15,21,22), as well as inclusion criteria. **B** Uniform Manifold Approximation and Projection (UMAP) plot representing 23 color-coded cell clusters derived from 71,459 CSF cells in the final cohort comprising 11 controls (Ctrl), 12 relapsing-remitting multiple sclerosis (RRMS), and 12 progressive multiple sclerosis (PMS) patients. **C** Dotplots depicting marker genes of cell clusters. Dot size encodes the percentage expressed, and the color code shows the average expression of the depicted genes. **D** Volcano plots depicting changes of cluster abundance in RRMS vs. PMS. Logarithmic two-fold change (Log2FC) of cell type abundance is plotted against negative logarithmic adjusted *p*-value for multiple hypotheses (pvalue_adj). The horizontal line visualizes the significance threshold (adjusted *p*-value 0.05). E Stacked bar plot of the proportion of cells in Ctrl, RRMS, and PMS. Abbreviations: Border-associated macrophages (BAM)-like cells, non-classical monocytes (ncMono), classical monocytes (cMono), conventional DC type 2 (cDC2), conventional DC type 1 (cDC1), plasmacytoid DC (pDC), B cells (Bc), Plasma cells (Plasma), natural killer (NK), double negative T cells (dnT), regulatory T cells (Treg), central memory T cells (TCM), effector memory T cells (TEM)

We newly collected CSF cells from 13 patients with PMS (6 PPMS, 7 SPMS), 9 RRMS patients, and 8 patients with idiopathic intracranial hypertension or other non-inflammatory diseases (Ctrl). In addition, we added 5 Ctrl samples and 5 RRMS data from previously published single-cell RNA sequencing (scRNA-Seq) studies from our group [15, 21, 22] (Suppl. Tab. 1; Methods). After excluding samples with fewer than 100 cells or ambiguous demultiplexing results (Methods), the final dataset included 11 Ctrl, 12 RRMS, and 12 PMS (5 PPMS, 7 SPMS) samples (Fig. 2A).

A minimum of 3 ml of fresh CSF was centrifuged, and the supernatant was frozen separately. The CSF cell pellet was resuspended in 900 µl cold recovery cell culture freezing medium (Thermo Fisher Scientific), gradually cooled to −80 °C, and transferred to liquid nitrogen for long-term storage. The CSF of 5 PMS patients (PPMS_6, SPMS_4-7, Suppl. Tab. 1) was processed directly after collection without the freezing step, as described in [15, 21, 22].

Ctrls were mainly diagnosed with idiopathic intracranial hypertension (IIH), one patient was diagnosed with a cognitive disorder, and one with normal pressure hydrocephalus (NPH). RRMS patients were diagnosed based on the revised McDonald criteria 2017 [17] that have experienced an active relapse. Sampling of the RRMS group was timed before initiation of immunomodifying therapies, whereas sampling of the PMS cohort was performed at later disease courses, resulting in 8 out of 12 PMS patients having already received a disease-modifying therapy (DMT) prior to sampling (Suppl. Tab. 1). One SPMS patient received CSF diagnostics within several weeks, thereby generating a biological replicate, which was grouped as patient SPMS_4 in the following analysis (Suppl. Tab. 1).

We performed the best possible matching for clinical data, sex, and age between the cohorts. The average age of the Ctrl cohort was 44 years, of the RRMS patients 42 years, and of the PMS patients 62 years (Suppl. Figure 2A). Exclusion criteria encompassed concurrent immunological comorbidities, specific conditions (e.g., pregnancy or breastfeeding and age under 18), severe concomitant infectious diseases, or artificial blood contamination in the CSF (> 200 RBCs/µL) [15]. All participants provided informed consent, and the studies were ethically approved by the relevant committees (AZ: 2015—522—f-S).

### Creating and sequencing single-cell libraries

Frozen CSF was thawed at 37 °C in complete growth medium (10% FBS in RPMI), washed, and then processed as described in [23], with the modification that cells were not sorted due to high cell loss observed during this step; fresh CSF was processed directly. To generate single-cell libraries, cell suspensions were loaded onto the Chromium Single Cell Controller with the Chromium Single Cell 5′ Library & Gel Bead Kit v.1.1/v2 (both from 10X Genomics) in adherence to the manufacturer's guidelines. The fresh PMS CSF samples were processed individually using the Single Cell 5’ Library Kit with a single index. Frozen CSF samples were pooled, ensuring that each disease stage and Ctrls were represented in each pool and that approximately the same number of cells per patient were used within the pool, resulting in a total of 3 pools that were processed using the Single Cell 5' Kit with dual index. Sample processing and library preparation followed the manufacturer's instructions and involved the use of AMPure beads (Beckman Coulter). Subsequently, sequencing was conducted for the fresh samples on a local Illumina NextSeq 500 using the High-Out 75-cycle kit with a 26—8—0—57 read setup or on a local Illumina NextSeq 2000 using the P3 100-cycle kit with a 28—8—0—91 read setup and for the frozen samples, the NextSeq 2000 using the P4 100-cycle kit with a 28—10—10—90 read setup.

### Demultiplexing of pooled scRNA-seq data

For demultiplexing of the pooled samples, we performed bulk RNA sequencing (RNA-seq) of peripheral blood mononuclear cells (PBMCs) from the corresponding patients, extracted single-nucleotide polymorphisms (SNPs) of the individual samples, and matched these to the scRNA-seq data, enabling assignment of individual cells to their donor of origin.

In detail, PBMCs were isolated from the patients' blood as previously described in [16, 24]. Subsequently, RNA was isolated from 100 µl PBMC using the Quick-RNA microprep kit (Zymo Research), followed by cDNA synthesis and library preparation using the SMART-Seq mRNA LP (with UMIs) Kit (Takara) according to the manufacturer's instructions. Sequencing was performed on the Novaseq X using the 1.5B 200-cycle kit in a 111—8—8—111 read configuration. SNPs from the RNA-seq data were identified using cellsnp-lite (mode 2b) with filtering criteria of minimum minor allele frequency (MAF) > 0.1 and minimum read count > 20 across chromosomes 1—22, X, and Y. These SNPs were then genotyped in CSF scRNA-seq data using cellsnp-lite (mode 1a) with the same quality thresholds, and only SNPs detected in both bulk and scRNA-seq were retained for demultiplexing. Cell-to-donor assignment was performed using demuxalot [25]. Cells assigned to multiple donors (doublets) and patients with inconclusive demultiplexing results were removed from downstream analysis.

### Preprocessing scRNA-seq data

The sequencing data underwent processing using Cell Ranger v8 (from 10X Genomics). Raw bcl files were demultiplexed employing cellranger mkfastq. Read alignments and transcript counting were carried out for each sample separately using Cell Ranger count with standard parameters. To ensure consistency, Cell Ranger aggr was utilized to equalize the number of confidently mapped reads per cell across all samples. The computations using Cell Ranger were performed on the High-Performance Computing (HPC) cluster PALMA II of the University of Muenster.

### scRNA-seq data analysis

The downstream analysis was carried out using *Seurat v5.1.0* in *R v4.5.1*, following the official tutorial. Briefly, the quality of each sample was inspected individually, and cells with low gene counts (< 200), excessively high gene counts (> 15,000), or high mitochondrial (> 7.5%), erythrocyte (> 5%), and ribosomal (> 50%) percentages were filtered out with individual thresholds. Removal of cell doublets was executed using *scDblFinder v1.22.0* default parameters, and patients with < 100 cells post-filtering were removed for downstream analysis. Subsequently, a total of 71,459 cells were retained for the analysis, with 2,042 average cells per sample and 1,088 median genes detected per cell (Suppl. Tab. 1). Data integration was performed using *SCTransform v2* normalization followed by Seurat’s reciprocal PCA workflow with 2,000 integration features and k.weight = 75.

Clusters were identified using Seurat's "FindNeighbors" and "FindClusters" functions (Louvain method, resolution 0.7) based on SCT embeddings. Uniform Manifold Approximation and Projection (UMAP) was run in Seurat using SCT embeddings. Differential expression of genes was computed using Seurat's "FindMarker" function (Wilcoxon rank-sum test, threshold for significant genes: avg_log2FC > 0.5, p_val_adj < 0.001). Clusters were annotated using *Azimuth v0.5.0* for initial automatic annotation, followed by manual refinement based on differentially expressed gene signatures and literature research. We identified three monocyte clusters (*CD14*, *FCGR3A*/CD16), including classical monocytes (cMono: *CD14* + + *, FCGR3A*/*CD16*) and two clusters resembling border-associated macrophages (BAM and preBAM: *CD14, CD16, C1QB, APOE, LYVE1, TREM2*). One of these two BAM-like clusters exhibited lower expression of canonical BAM markers. We therefore performed trajectory analysis of the monocyte population (cMono, BAM, preBAM) using *Slingshot v2.16.0* [26] after subsetting the respective clusters and identified these clusters in an intermediate position between cMono and BAMs (Suppl. Figure 2 C). We therefore termed this population 'preBAM,' suggesting a transitional state towards BAM differentiation. Three dendritic cell (DC) clusters were separated into conventional DC type 2 (cDC2: *FCER1A, CD1C*), conventional DC type 1 (cDC1: *XCR1*, *CLEC9A*), and plasmacytoid DC (pDC: *LRRC26, CLEC4C*). Lymphocytes were divided into two B lineage clusters – B cells (Bc: *CD19, MS4A1, CD79A*) and plasma cells (Plasma: *CD79A*, *IGHG1*) –, one natural killer cell cluster (NK: *GNLY*, *NCAM1), and* 12 T cell clusters (Tc: *CD3E*). T cell clusters were separated into regulatory T cells (Treg: *IKZF2, FOXP3*), CD4^+^ cells, and CD8^+^ cells (*CD8A*) and further subdivided into effector memory (TEM: *GZMA, GZMK*), central memory (TCM: *IL7R, CCR7, CD27*), naive (*SELL*), and activated (*CD69*) phenotypes. Additionally, two clusters showed elevated expression of ribosomal, mitochondrial, and long non-coding RNA genes (e.g., *RPS4Y1, ENSG00000273149*), indicating stress-related cell states.

Cluster abundance was determined with *propeller* [27], included in *speckle v1.8.0*, following the official tutorial. A logit transformation of cell type proportions, followed by linear modeling to test for compositional differences while accounting for sample variability, was used. Additional confounder adjustment was applied for sex and age. A t-test was carried out with the propeller.ttest function with a robust variance estimation. *P*-values were adjusted for multiple testing using the Benjamini–Hochberg method.

Over-representation GO term analysis was performed using the package *clusterProfiler* v4.16.0 using the *enrichGO()* function [28] on significant up- (avg_log2FC > 0.5, p_v_adj < 0.001) and down-regulated genes (avg_log2FC < 0.5, p_v_adj < 0.001).

### Expanded scRNA-seq cohort

We expanded our scRNA-seq analysis by integrating publicly available CSF scRNA-seq datasets from age-stratified Ctrls and AD patients [29, 30]. Specifically, raw data from Piehl et al. [29] and Gate et al. [30] were reprocessed using the same pipeline as for our cohort (methods: *Preprocessing scRNA-seq Data* and *scRNA-seq data analysis).* We selected data from eight AD patients, 12 Ctrls < 65 years (“young”), 12 Ctrls aged 65-75 years (“mid”), and nine Ctrls > 75 years (“old”), and integrated them with our dataset (methods: *scRNA-seq data analysis)*. The combined dataset was clustered at a resolution of 0.68, resulting in 23 distinct clusters, and original cell annotations were transferred using Seurat’s “FindTransferAnchors” and "TransferData" functions. Cell abundance and DE analysis were performed as described (methods: *scRNA-seq data analysis).*

For condition-specific BAM analysis, BAM-like cells were subset by disease condition (Ctrl, RRMS, PPMS, SPMS, AD) and normalized using SCTransform v2. Condition-specific markers were identified with Seurat's "FindMarkers" function (min.pct = 0.25, logfc.threshold = 0.25) after removing mitochondrial, ribosomal, and non-coding genes. The top 20 genes per condition were visualized in a scaled heatmap (pheatmap), and overrepresentation GO term analysis was performed using the package *clusterProfiler v4.16.0 *and the *enrichGO()* function [28, 31].

### Enzyme-linked immunosorbent assay

Soluble TREM2 (sTREM2) levels were measured in the CSF supernatant obtained from Münster, Germany, and Lausanne, Switzerland, biobanks for 25 Ctrls, 24 RRMS, 37 PMS (20 PPMS and 17 SPMS), and 18 AD patients using the *Human TREM2 SimpleStep ELISA Ki*t (abcam, ab224881) according to the manufacturer's instructions. Briefly, thawed CSF supernatant was analyzed in duplicate using 50 µl per well, and absorbance was recorded with an Infinite M200 Pro plate reader (Tecan).

## Results

### Flow cytometry reveals a relative expansion of monocytes in progressive multiple sclerosis

We initially studied cellular alterations in the CSF of PMS patients using retrospective analysis of existing multi-parameter flow cytometry data. We collected CSF flow cytometry data of 74 non-inflammatory controls (Ctrl; 46 females, 28 males), 169 relapsing–remitting multiple sclerosis (RRMS) patients (124 females, 45 males), and 56 progressive MS (PMS) patients (33 females, 23 males) (Suppl. Tab. 1, Fig. 1 A). Out of the 56 PMS patients, 30 were diagnosed with primary progressive MS (PPMS; 18 females, 12 males) and 26 with secondary progressive MS (SPMS; 15 females, 11 males). As expected, PMS patients were older than RRMS and Ctrl patients (Suppl. Figure 1 A), and across all cohorts, there were more female than male patients (Suppl. Figure 1 B). PMS patients exhibited a higher expanded disability status scale (EDSS) score and a longer disease duration, while RRMS patients had more relapses in the last year prior to sample collection (Suppl. Figure 1 C-E).

When systematically comparing flow cytometry data of CSF from RRMS patients to Ctrl, we replicated the characteristic increases of plasma cells, B cells, total cell count, lymphocytes, and activated (HLA-DR^+^) T cells [15, 22, 32, 33], while the percentage of monocytes was significantly decreased in RRMS patients compared to Ctrls (Suppl. Figure 1 F). Similar cellular alterations were detected when comparing the CSF of PMS patients to Ctrls, albeit less pronounced (Suppl. Figure 1 G). When directly comparing PMS with RRMS patients, the relative proportion of total monocytes and NKT was increased in PMS, while the cell count, plasma cells, total lymphocytes, and B cells significantly decreased in PMS (Fig. 1 B, Suppl. Figure 1 H). These findings remained consistent after controlling for age and sex (Suppl. Figure 1 I).

When comparing with clinical information, we observed a positive correlation between the percentage of monocytes in the CSF and the EDSS of MS patients (Fig. 1 C), while monocyte percentage negatively correlated with both radiological and clinical disease activity (Fig. 1 D-E). Relative CSF monocyte abundance is thus higher in PMS and is associated with surrogate markers of MS disease progression in the absence of active inflammation.

### Single-cell transcriptomic characterization of CSF cells in progressive multiple sclerosis

Therefore, we aimed to more deeply characterize CSF leukocyte differences (while focusing on myeloid cells) in RRMS vs. PMS by performing single-cell transcriptomics of CSF cells of newly recruited donors across the MS disease spectrum and Ctrls (Methods). The final dataset included 11 Ctrl, 12 RRMS, and 12 PMS (5 PPMS, 7 SPMS) samples, resulting in a total of 71,459 high-quality single-cell transcriptomes (subsequently denoted as ‘cells’ for simplicity) (Fig. 2A, Suppl. Figure 2 A-B, Methods). Unbiased clustering resulted in 23 individual clusters (Fig. 2 B). Based on the expression levels of marker genes (Fig. 2 C, Suppl. Tab. 2), we identified three monocyte clusters (*CD14*, *FCGR3A*/CD16), including classical monocytes (cMono: *CD14* + + *, FCGR3A*/*CD16*) and two clusters resembling border-associated macrophage-like cells (BAM and preBAM: *CD14, CD16, C1QB, APOE, LYVE1, TREM2*). One of these two BAM-like clusters exhibited lower expression of canonical BAM markers and occupied an intermediate position between cMono and BAMs in trajectory analysis (Suppl. Figure 2 C). We therefore termed this population 'preBAM,' suggesting a transitional state towards BAM-like cell differentiation. Moreover, we found three dendritic cell (DC) clusters and 15 lymphocyte clusters. Lymphocytes were divided into two B lineage clusters, one natural killer cell cluster*,* and 12 T cell clusters (Methods, Fig. 2 C, Suppl. Tab. 2). We thus reconstructed the known composition of CSF cells.

Of note, we included data from both fresh and frozen CSF cells (Methods) and observed no major differences in overall cell type distribution between the two approaches (Suppl. Figure 2D), in accordance with previous studies [23, 34]. However, one T cell cluster (Tc_stressed) appeared specific to a single processing method and was therefore disregarded in downstream analyses due to its likely technical origin (Suppl. Figure 2 D-E).

### CSF myeloid cells respond to progressive MS

Next, we aimed to identify compositional changes by comparing MS vs. Ctrl samples (Suppl. Figure 2 F-G). As previously described, proportions of B cells and CD8^+^TEM cells were significantly higher in the CSF of RRMS patients compared to Ctrls [15, 22], whereas BAM-like cells, cMono, and cDC2 were reduced in RRMS (Suppl. Figure 2F) [33]. None of the clusters were significantly increased or decreased in PMS patients compared with the Ctrl cohort, likely due to a lack of statistical power or lower effect sizes. Nevertheless, we found a non-significant trend towards expansion of B cells, CD8^+^ TEM cells, and preBAM in PMS, while activated T cells, cDC2, and plasma cells tended to be more abundant in Ctrls (Suppl. Figure 2 G).

Next, we directly compared PMS with RRMS. BAM-like cells showed a significant increase in PMS, while B cells were significantly more abundant in RRMS (Fig. 2 D). This relative increase in innate/myeloid cell populations (e.g. BAM-like cells) in PMS was in line with our flow cytometry findings. Monocytes, predominantly of the BAM-like phenotype, were lost from the CSF in RRMS but relatively enriched in PMS, whereas B cells were more abundant in both MS forms compared with Ctrls (Fig. 2 E). A relative increase of BAM-like cells is thus a unique feature of CSF in PMS.

### BAM-like cells in PMS are primed for sustained antigen presentation

**Fig. 3:**
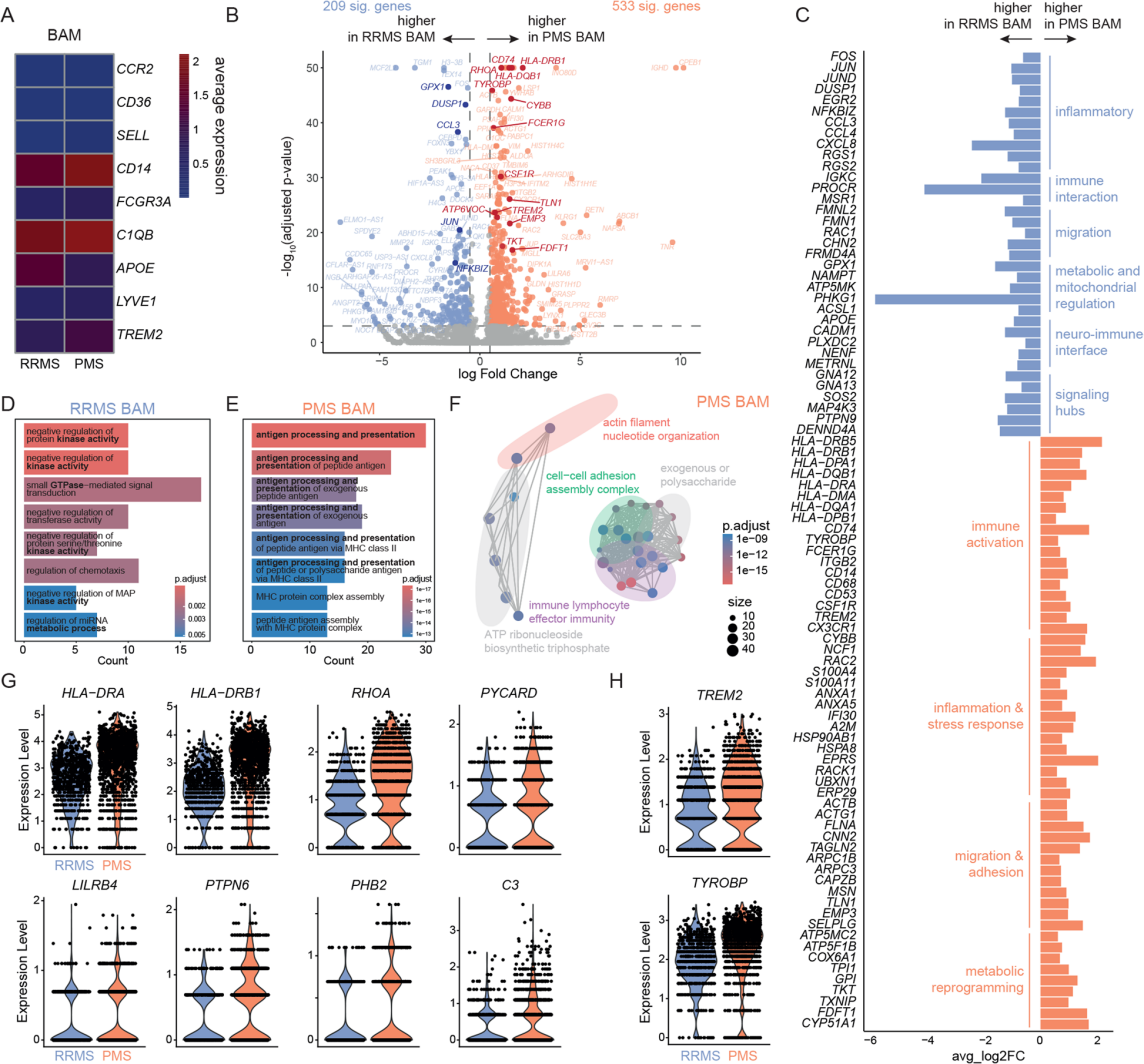
Myeloid cells in progressive multiple sclerosis cerebrospinal fluid acquire a border-associated macrophage-like phenotype with enhanced antigen-presenting potential. **A** Heatmap visualizing the average log-normalized expression of selected monocyte and border-associated macrophage (BAM) marker genes in cerebrospinal fluid (CSF) BAM-like cells derived from relapsing–remitting multiple sclerosis (RRMS) patients and patients with progressive multiple sclerosis (PMS). Color intensity reflects relative expression (dark blue = low expression, dark red = high expression). **B** Volcano plot of differentially expressed (DE) genes in RRMS-derived and PMS-derived BAM-like cells. Significant genes upregulated in PMS-derived BAM-like cells are shown in red, and those upregulated in RRMS-derived BAM-like cells in blue. Highlighted genes include immediate-early and inflammatory mediators, regulators of metabolism and mitochondrial function, components of GTPase and kinase signaling pathways, antigen-presentation molecules, activation and phagocytosis markers, stress-response genes, migration/adhesion molecules, and genes related to glycolysis, oxidative phosphorylation, and cholesterol biosynthesis pathways. Logarithmic (log) fold change is plotted against negative log adjusted *p*-value for multiple hypotheses. The horizontal line visualizes the significance threshold (adjusted *p*-value 0.05). **C** Bar plot showing genes with higher expression in RRMS-derived BAM-like cells (blue) or PMS-derived BAM-like cells (orange), grouped by functional categories. The x-axis indicates average log two-fold change (avg_log2FC). **D**-**F** Gene ontology (GO) term enrichment analysis of DE genes (average log2 fold change > 0.5, adjusted *p*-value = 0.001) of (**D**) RRMS-derived and (**E**)-(**F**) PMS-derived BAM-like cells using the package *ClusterProfiler.* **G**-**H** Violin plots of DE genes of BAM-like cells in PMS (red) vs. RRMS (blue) of genes associated with (**G**) antigen presentation, leukocyte regulation, immune effector processes, and **H** neurodegenerative mechanisms. All genes depicted are significant (average log2 fold change > 0.5, adjusted *p*-value = 0.001)

We next sought to more deeply understand the cellular phenotype of the BAM-like cell cluster. In accordance with our annotation, BAM-like cells from both PMS and RRMS patients exhibited a border-associated macrophage phenotype, characterized by the absence of classical monocyte markers (*CCR2, CD36,* CD62L/*SELL*) and the expression of established BAM markers (*C1QB, APOE, LYVE1, TREM2*) (Fig. 3 A). Notably, *APOE* expression was slightly higher in RRMS-derived BAM-like cells, whereas *TREM2* and *LYVE1* expression was elevated in PMS-derived BAM-like cells, indicating not only differences in cell abundance but also disease stage-associated transcriptional changes within this population.

In fact, differential expression (DE) analysis identified 533 significantly upregulated genes in PMS-derived BAM-like cells and 209 in RRMS-derived BAM-like cells after removal of mitochondrial, ribosomal, zinc finger, and long non-coding RNA genes (Fig. 3 B; Methods, Suppl. Tab. 3). In detail, RRMS-derived BAM-like cells showed higher expression of immediate-early genes and inflammatory genes (*JUN, DUSP1, NFKBIZ, CCL3, CCL4*), along with genes involved in metabolic and mitochondrial regulation (*GPX1, NAMPT, ACSL1*) and components of GTPase and kinase signaling pathways (*GNA13, GNAQ, MAP4K3, RGS2*) (Fig. 3 B-D). This transcriptional profile suggests a “ready state”, characterized by dynamic signaling capacity and rapid remodeling in response to acute inflammatory cues. In contrast, PMS-derived BAM-like cells predominantly expressed antigen-presenting genes (*HLA-DRB5, HLA-DRB1, HLA-DQB1*), activation and phagocytosis markers (*TYROBP, FCER1G, CD68, CSF1R*), genes associated with stress response (*CYBB, NCF1, RAC2, HSP90AB1),* and migration and adhesion molecules (*MSN1, TLN1, EMP3*). They also exhibited a hybrid metabolic state from glycolysis (*TPI1, GPI, TKT*), oxidative phosphorylation (*ATP6VOC, ATP5MC2, COX6A1*), and cholesterol biosynthesis (*FDFT1, CYP51A1*) (Fig. 3 B-C). Collectively, this transcriptional profile is consistent with a chronic, non-resolving inflammatory phenotype and a BAM-like cell population primed for chronic antigen presentation and persistent immune signaling. These findings were further supported by overrepresentation enrichment and cell-cell interaction analysis, which highlighted PMS-derived BAM-like cells as antigen-presenting cells (*HLA-DRA, HLA-DRB1*) (Fig. 3 E–G, Suppl. Figure 2 H) with enrichment for pathways involved in the positive regulation of leukocyte cell-cell adhesion (*RHOA, PYCARD, LILRB4*) and immune effector processes (*PTPN6, PHB2, PYCARD, C3*) (Fig. 3 E–G). Chronically activated BAM-like cells with antigen-presenting capacity thus characterize the CSF in PMS.

Next to their chronic antigen-presenting phenotype, PMS-derived BAM-like cells expressed genes previously identified in disease-associated microglia/macrophages (DAMs) in Alzheimer’s disease (AD) [35] (*TREM2, TYROBP*) (Fig. 3 A-C, H), suggesting potential links between disease progression in MS and neurodegeneration.

### Distinct myeloid signatures in SPMS reflect disease progression

**Fig. 4:**
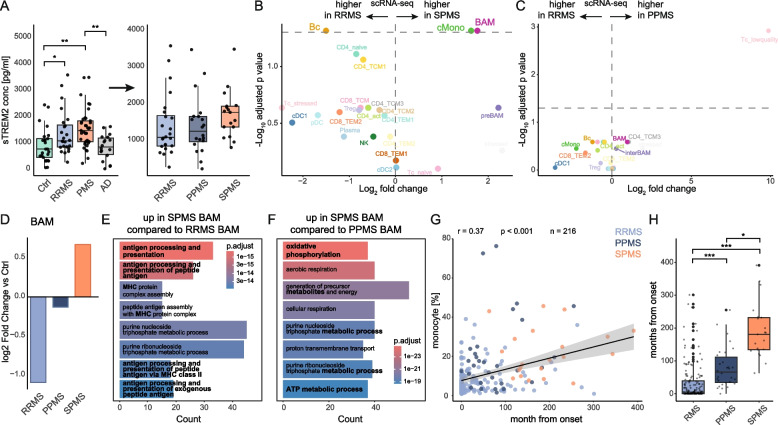
TREM2 marks progressive multiple sclerosis. **A** Dot plots depicting sTREM2 concentration in the cerebrospinal fluid (CSF) supernatant measured by enzyme-linked immunosorbent assay (ELISA). Comparison between controls (Ctrl), relapsing–remitting multiple sclerosis (RRMS), progressive multiple sclerosis (PMS), and Alzheimer's disease (AD) patients. The right panel shows sTREM2 levels in the different MS forms, and PMS is subdivided into primary progressive multiple sclerosis (PPMS) and secondary progressive multiple sclerosis (SPMS). pvalue <.05 = *; <.01 = **; <.001 = ***. **B**-**C** Volcano plots depicting changes in cell cluster abundance in (**B**) SPMS vs. RRMS and (**C**) PPMS vs. RRMS in the single-cell transcriptomic dataset. Logarithmic twofold change (Log2FC) of cell type abundance is plotted against negative logarithmic adjusted *p*-value for multiple hypotheses (pvalue_adj). The horizontal line visualizes the significance threshold (adjusted *p*-value 0.05). **D** Bar plot of log two-fold changes in border-associated macrophage-like (BAM) cluster abundance in the diseased state (RRMS, PPMS, and SPMS) compared to Ctrl. Bars above zero indicate increased abundance, and bars below zero indicate decreased abundance relative to Ctrl. **E**–**F** Gene ontology term enrichment analysis of differentially expressed (DE) genes (average log2 fold change > 0.5, adjusted *p*-value = 0.001) of SPMS-derived BAM-like cells compared to (**E**) RRMS-derived and (**F**) PPMS-derived BAM-like cells using the package *ClusterProfiler.* **G** Correlation analysis between the percentage of monocytes (flow-cytometry data) and the disease duration (months from onset). Data are shown as correlation plots with a linear regression line (solid line) and with its confidence interval in grey. Dots are colored by disease stage (RRMS—light blue, PPMS—dark blue, SPMS—orange). The Pearson correlation coefficient (r), the *p*-value (p), and the number of included patients (n) are depicted above the respective plots. **H** Dot plot depicts the disease duration (months from onset) of the flow cytometry cohort split by RMS, PPMS, and SPMS. pvalue <.05 = *; <.01 = **; <.001 = ***

Given the upregulation of DAMs-associated features, we decided to next focus on TREM2, a lipid-metabolizing molecule involved in tissue repair [36–38], one of the defining features of neurodegeneration-associated microglia [35], and both a potential biomarker and therapeutic target in neurodegenerative diseases [36, 39]. We therefore measured soluble TREM2 (sTREM2) in the CSF supernatant of MS patients with different disease courses as well as in IIH Ctrls and in patients with AD (25 Ctrl, 24 RRMS, 37 PMS (20 PPMS, 17 SPMS), 18 AD) using enzyme-linked immunosorbent assay (ELISA). sTREM2 concentration was significantly higher in MS — both in RRMS and in PMS — compared to Ctrls and AD (Fig. 4 A). After subsetting PMS into SPMS and PPMS, we observed a progressive increase in sTREM2 across the MS continuum (Fig. 4A).

Despite not reaching statistical significance for sTREM2 in a comparison within the MS continuum, we wondered if it would shift from dominating innate immune responses in RRMS patients toward more myeloid pro-degenerative responses in PMS. We therefore focused more specifically on the contribution of myeloid cells across the progressive subtypes of MS. Compared with RRMS, SPMS was characterized by a significant increase in BAM-like cells and cMonos and a reduction of B cells, whereas PPMS showed no noteworthy cell abundance changes compared with RRMS (Fig. 4 B-C). BAM-like cells were reduced in RRMS, slightly decreased in PPMS, and markedly elevated in SPMS, indicating re-emergence during disease progression (Fig. 4 D). SPMS-derived BAM-like cells showed increased expression of genes associated with antigen presentation and activation compared with RRMS and PPMS (Fig. 4 E–F). Moreover, CSF monocyte abundance correlated positively with disease duration, and SPMS patients exhibited significantly longer disease duration than PPMS and RRMS patients (Fig. 4 G-H). These findings suggest SPMS as a distinct, myeloid-dominant disease stage characterized by the activation and relative expansion of BAM-like cells in the CSF.

### Aging and progressive MS induce divergent cellular signatures in CSF

Given the unavoidable age difference between RRMS and PMS patients (Suppl. Figure 1 A, Suppl. Figure 2 A, Suppl. Figure 3 A), we next investigated the impact of age on the observed CSF myeloid alterations. In the flow cytometry cohort, the relative increase in monocytes in PMS remained significant after adjustment for age and sex (Suppl. Figure 1 I), and CSF monocyte frequency did not correlate with age in Ctrls (Suppl. Figure 3 B). Because the single-cell transcriptomic cohort lacked age overlap between RRMS and SPMS patients, and therefore age-adjustment would also remove substantial parts of the disease-stage signal, we integrated publicly available CSF single-cell transcriptomic datasets from age-stratified Ctrls and AD patients (Methods, Suppl. Figure 3C-E) [29, 30]. In this expanded cohort, BAM abundance and TREM2 expression in Ctrls were not associated with age (Suppl. Figure 3 F), whereas TREM2 expression remained highest in PMS, particularly in SPMS (Suppl. Figure 3 G). Consistently, the increase in sTREM2 in SPMS remained robust in both age-adjusted and age-matched subset analyses of the ELISA cohort (Suppl. Figure 3 H-J). Together, these findings argue against aging alone as the main driver of the observed myeloid alterations.

### SPMS-derived BAM-like cells share neurodegenerative features with AD-associated myeloid cells

Based on the DAM-like transcriptional signature of BAM-like cells in PMS, we hypothesized that there might be a link between mechanisms of disease progression in MS and in neurodegenerative diseases. To address this, we expanded our cohort to a canonical neurodegenerative disease, leveraging publicly available Ctrl and AD CSF single-cell transcriptomic datasets from Gate et al. [30] and Piehl et al. [29] (Methods).

**Fig. 5:**
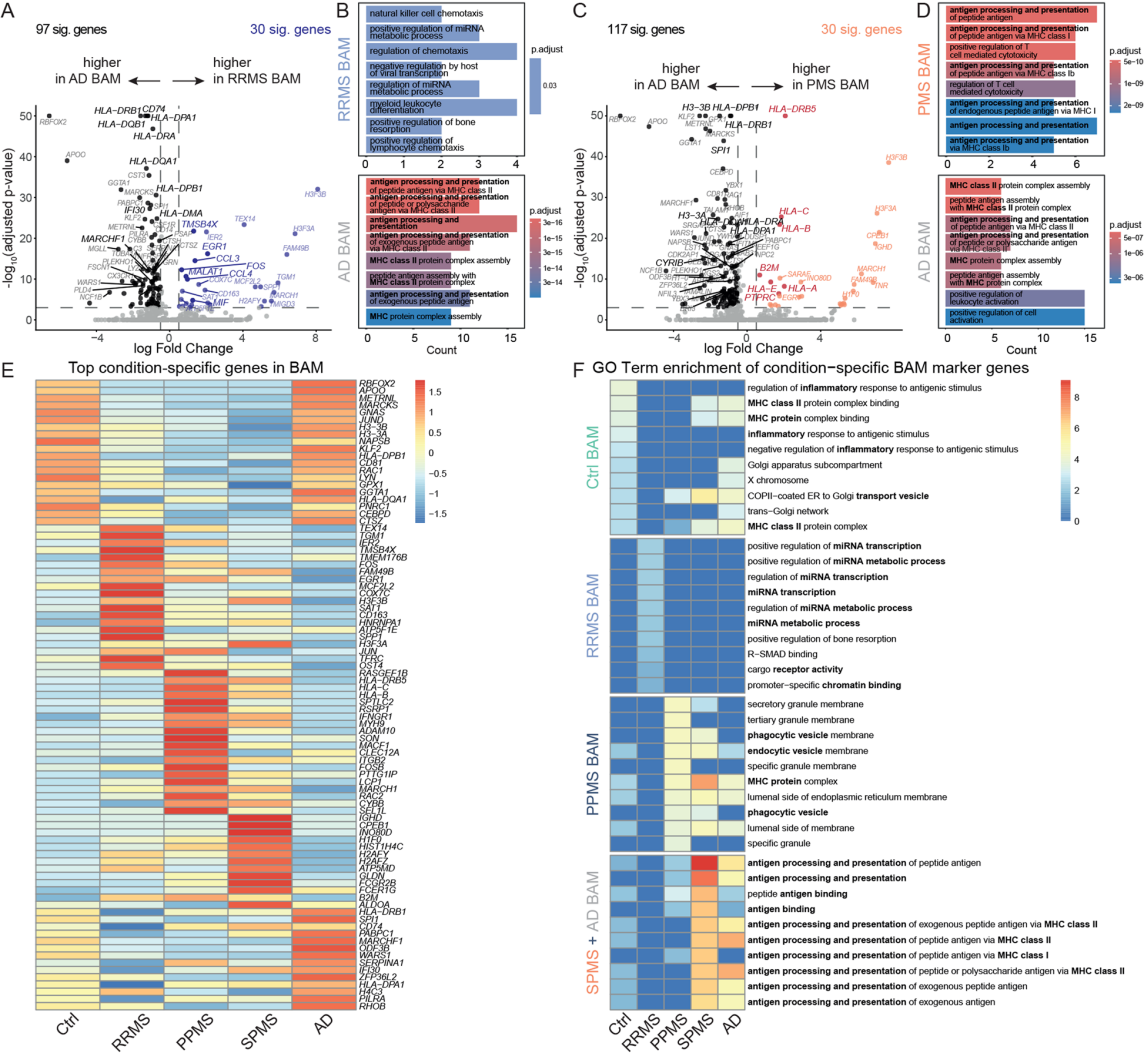
SPMS-derived BAM-like cells exhibit transcriptional similarities with AD-associated myeloid states. **A** and **C** Volcano plots of differentially expressed (DE) genes in (**A**) AD- vs RRMS-derived BAM-like cells and in (**C**) AD-derived vs PMS-derived BAM-like cells. Significant genes upregulated in RRMS-derived BAM-like cells are shown in blue, those upregulated in RRMS-derived BAM-like cells in black, and those upregulated in PMS-derived BAM-like cells in red. Highlighted genes are associated with the depicted pathways in (**B**) and (**D**). Logarithmic (log) fold change is plotted against negative log adjusted *p*-value for multiple hypotheses. The horizontal line visualizes the significance threshold (adjusted *p*-value 0.05). (**B**) and (**D**) Gene ontology (GO) term enrichment analysis of DE genes (average log2 fold change > 0.5, adjusted *p*-value = 0.001) of (**B**) RRMS- and AD-derived BAM-like cells and of **(D)** PMS- and AD-derived BAM-like cells using the package *ClusterProfiler.* **E** and **F** Heatmaps visualizing (**E**) average expression of condition-specific BAM-like cell marker genes and (**F**) enriched GO term pathways in BAM-like cells in the depicted conditions

Differential expression analysis showed that, compared with RRMS-derived BAM-like cells, AD-derived BAM-like cells were characterized by upregulation of genes related to antigen presentation (Fig. 5 A-B). Notably, PMS-derived BAM-like cells exhibited an even stronger antigen-presenting signature than AD-derived BAM-like cells (Fig. 5 C-D).

To further characterize BAM-like signatures across conditions, we performed condition-specific differential expression analysis followed by GO-term enrichment analysis on the top 20 DE genes (Methods). This revealed the strongest transcriptional overlap between SPMS- and AD-derived BAM-like cells, with shared enrichment of antigen presentation pathways, particularly MHC-mediated antigen presentation (Fig. 5 E–F). While Ctrl-derived BAM-like cells showed baseline immune sensing, RRMS-derived BAM-like cells were enriched for transcriptional remodeling, and PPMS-derived BAM-like cells showed enrichment of phagocytosis-related pathways, both SPMS- and AD-derived BAM-like cells were dominated by MHC-driven antigen presentation programs.

Taken together, these findings indicate that SPMS-derived BAM-like cells in the CSF share features with neurodegenerative diseases.

## Discussion

In this study, we combined protein and single-cell transcriptomic CSF cell immunophenotyping to delineate immune populations associated with disease progression in MS. We found that PMS was characterized by an expansion of myeloid cell populations in CSF, in particular BAM-like cells. Such BAM-like cells derived from RRMS patients exhibited signs of acute inflammatory response, whereas PMS-derived BAM-like cells were primed for chronic antigen presentation and persistent immune signaling. This was marked by elevated *TREM2 *expression, indicating a self-sustaining smoldering inflammation and neurodegenerative process in PMS [40, 41], and potential mechanistic similarities with neurodegenerative diseases.

The CNS harbors different tissue-resident macrophages: parenchymal microglia and BAMs [42, 43]. BAMs occupy the meninges, perivascular spaces, and choroid plexus and display distinct, tissue-adapted phenotypes that contribute to health and disease [44]. In MS, BAMs are linked to CNS autoimmunity and pathology, potentially by providing antigen-presenting function [45].

Our data revealed a shift of CSF immune responses from B cell-dominated [15, 46] toward a myeloid-enriched compartment with a BAM-like phenotype in PMS. Notably, this myeloid expansion and activation was most pronounced in SPMS, which exhibited enhanced antigen presentation and metabolic reprogramming in BAM-like cells relative to RRMS and PPMS. This pattern aligns with growing evidence that PMS is at least partially driven by myeloid-mediated pathology. Chronic active (“rim”) lesions, linked to disability progression [7], exhibit pronounced microglial and macrophage activation [11, 47]. Similarly, in chronic PMS, interface macrophages and microglia accumulate along the CSF/ependymal border, with MHC class II upregulation correlating with neuronal loss [48], highlighting the role of interface myeloid cells in sustaining CNS-compartmentalized pathology. Remarkably, we here defined that a similar signature was mirrored in the CSF.

The transcriptional program identified in CSF BAM-like cells in PMS, characterized by elevated TREM2, enhanced antigen presentation, and metabolic reprogramming, resembles disease-associated microglia (DAMs) and monocyte-derived disease-inflammatory macrophages (DIMs) described in aging and neurodegeneration [41, 49]. Interestingly, a recently published study by Fissolo et al. [50] reported negative findings for sTREM2 detected in the blood as a long-term biomarker in PPMS. This contrasts with our observation of elevated CSF sTREM2 in SPMS, reflecting most likely both compartment-specific differences (blood vs. CSF) and subtype-specific effects (PPMS vs. SPMS). These findings align with neuropathological studies showing increased *TREM2* expression in microglia/macrophages within chronic active SPMS lesions [51]. Chronic DAM activation with aging or in neurodegenerative disorders such as AD drives inflammation, synaptic loss, and brain atrophy [52–54]. A similar pattern is observed in parenchymal broad-rim lesions, myeloid-rich rims detectable via TSPO-PET, which correlates with rapid clinical progression and resembles neurodegenerative myeloid signatures [47], highlighting the link between border-proximal myeloid activity and CNS-compartmentalized neurodegeneration.

Age-related alterations in the CSF have been described previously, primarily affecting the lymphoid population [20, 55]. Whether TREM2-expressing DIM/DAMs are linked to aging and neurodegeneration remains controversial [35, 41, 56, 57]. While age may contribute to the observed phenotype in PMS, our analysis indicated that aging alone is unlikely to explain the CSF changes in PMS. Instead, the relative expansion of BAM-like cells, their DAM-like phenotype, and the increase of TREM2 levels in PMS seem to reflect disease progression and neurodegenerative processes beyond age-related effects.

Together, these observations may support a therapeutic shift from targeting predominantly adaptive, B-cell-driven inflammation to addressing myeloid-mediated neurodegeneration. Bruton tyrosine kinase inhibitors (BTKi) can, on the one side, modulate B cell receptor signaling, thereby affecting antibody production, cytokine secretion, and B cell maturation, and on the other side, they engage CNS-resident microglia/macrophage pathways, and promoting anti-inflammatory reprogramming [14, 58]. Emerging data from randomized clinical trials indicate that BTKi affects progression independent of overt inflammatory activity [13]. It remains intriguing to speculate whether SPMS patients, with the most pronounced BAM-like activation (Fig. 3) and evidence of B-cell/plasma-cell maturation (Suppl. Figure 4), may represent an optimal group for early myeloid-targeted intervention and whether CSF BAM profiling could serve as a corresponding biomarker.

Our study has limitations: The MS cohorts are heterogenous, and we have grouped the PPMS and SPMS cohorts for some analyses. We did not perform longitudinal analysis, and CSF sampling was performed at different stages of disease: sampling of the RRMS group was timed before initiation of immunomodifying therapies, whereas sampling of the PMS cohort was performed at later disease courses (Suppl. Tab. 1). While this approach is ethically unavoidable given the clinical circumstances, it may be associated with potential effects of previous or ongoing treatments, as well as with undetectable disease activity or incomplete clinical data from the patient’s medical history. Nevertheless, it currently represents the best possible way to capture both disease spectra due to the assumption of the coexistence of partially progressive underlying mechanisms during all forms of MS. Despite these limitations, our study provides a valuable snapshot of immunological changes in CSF across the entire disease spectrum and can serve as a foundation for further complementary research with strong biomarker potential.

Collectively, we here successfully mapped the transcriptional landscape in the CSF cells in PMS. Expansion of BAM-like CSF myeloid cells in PMS hints towards potential diagnostic and therapeutic targets for neurodegenerative processes in MS in the future.

## Supplementary Information

Supplementary Material 1.

Supplementary Material 2.

Supplementary Material 3.

Supplementary Material 4.

Supplementary Material 5.

Supplementary Material 6.

Supplementary Material 7.

## Data Availability

The datasets supporting the conclusion of this article will be made available in the Gene Expression Omnibus (GEO) repository. We generated an interactive ShinyApp to explore the dataset without requiring bioinformatics expertise (https://osmzhlab.uni-muenster.de/shiny/cerebro_PMS).

## Abbreviations

AD: Alzheimer’s disease
AUC: Area under the curve
BAM: Border-associated macrophages
Bc: B cells
BTKi: Bruton’s tyrosine kinase inhibitor(s)
cDC: Conventional dendritic cell
cDC1: Conventional dendritic cell type 1
cDC2: Conventional dendritic cell type 2
cMono: Classical monocytes
CNS: Central nervous system
Ctrl: Controls
CSF: Cerebrospinal fluid
DAM: Disease-associated microglia/macrophages
DC: Dendritic cell
DE: Differentially expressed
DMT: Disease-modifying therapy/therapies
DIM: Disease-inflammatory macrophage(s)
dnT: Double-negative T cells
EDSS: Expanded Disability Status Scale
ELISA: Enzyme-linked immunosorbent assay
GEO: Gene Expression Omnibus
GO: Gene Ontology
Gd +: Gadolinium-enhancing (lesions) positive
HLA-DR: Human leukocyte antigen DR isotype
IIH: Idiopathic intracranial hypertension
MAF: Minor allele frequency
MRI: Magnetic resonance imaging
MS: Multiple sclerosis
ncMono: Non-classical monocytes
NK: Natural killer (cell)
NKT: Natural killer T (cells)
NPH: Normal pressure hydrocephalus
OCB/OCBs: Oligoclonal band(s)
pDC: Plasmacytoid dendritic cell
PCA: Principal component analysis
PIRA: Progression independent of relapse activity
Plasma: Plasma cells
PMS: Progressive multiple sclerosis
PPMS: Primary progressive multiple sclerosis
RBC/RBCs: Red blood cell(s)
RRMS: Relapsing–remitting multiple sclerosis
scRNA-seq: Single-cell RNA sequencing
SNPs: Single-nucleotide polymorphisms
SPMS: Secondary progressive multiple sclerosis
SSC: Side scatter
SSCint: Side-scatter intensity
sTREM2: Soluble TREM2
Tc: T cells
TCM: T central memory (cells) / central memory T cells
TEM: T effector memory (cells) / effector memory T cells
Treg: Regulatory T cells
TREM2: Triggering receptor expressed on myeloid cells 2
UMAP: Uniform Manifold Approximation and Projection
