# Supplementary figures and images for: A CSF disease-associated macrophage signature defines progressive multiple sclerosis
Source: J Neuroinflammation. 2026 May 13;23:156. doi: 10.1186/s12974-026-03861-9 (PMC13179637; doi:10.1186/s12974-026-03861-9)

Supplemental Figure 1

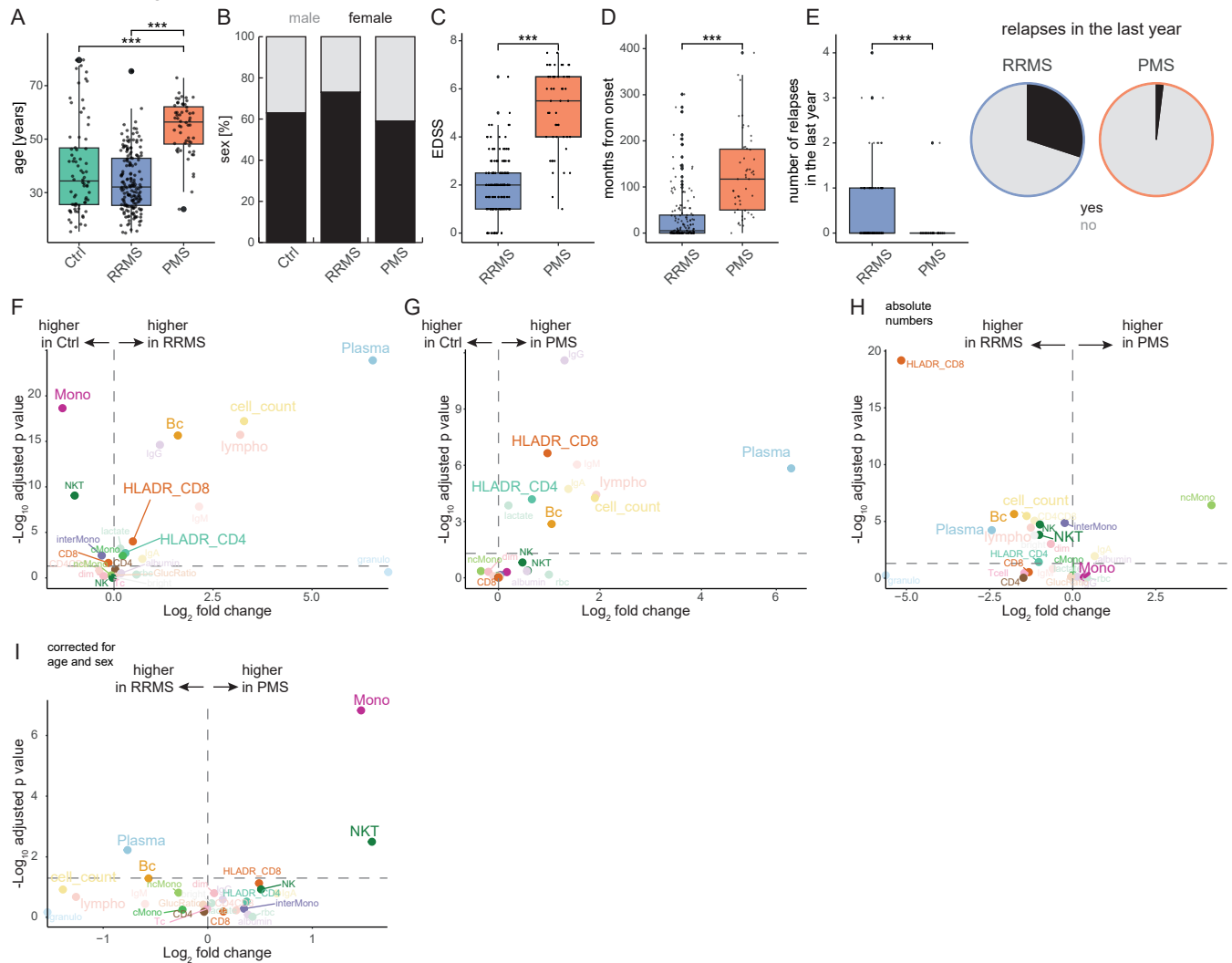

Supplement: Supplementary file 4 — Supplementary Material 4. [file 12974_2026_3861_MOESM4_ESM.pdf]

Suppl. Figure 2

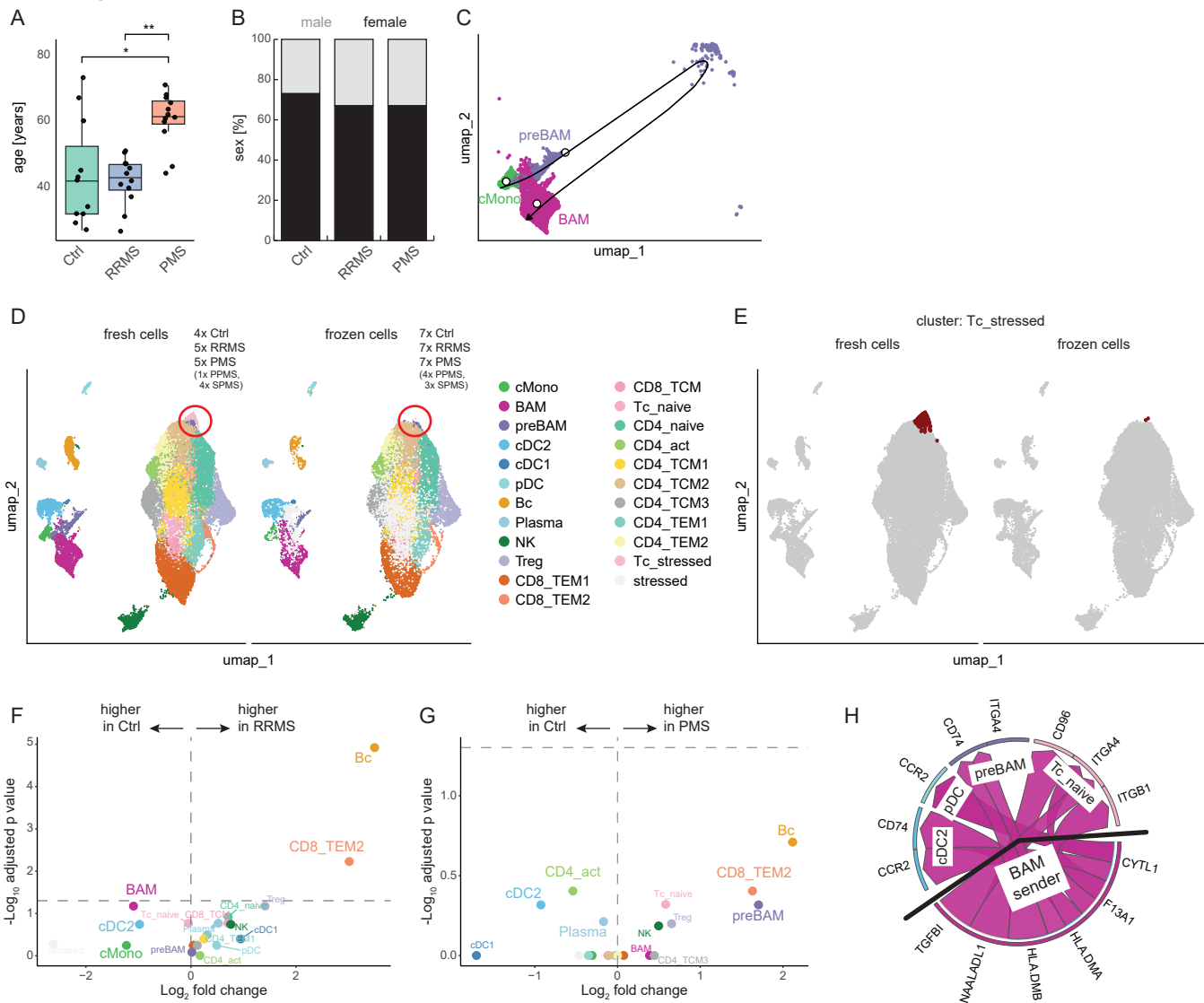

Supplement: Supplementary file 5 — Supplementary Material 5. [file 12974_2026_3861_MOESM5_ESM.pdf]

Suppl. Figure 3

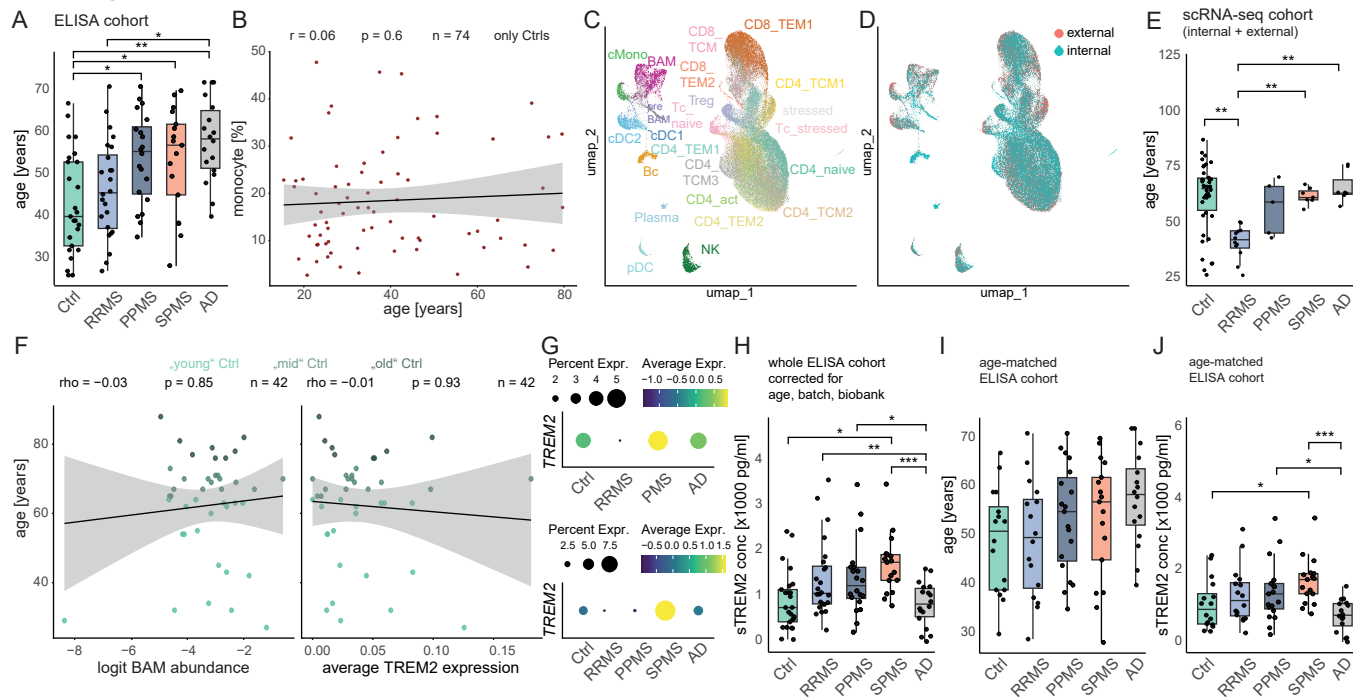

Supplement: Supplementary file 6 — Supplementary Material 6. [file 12974_2026_3861_MOESM6_ESM.pdf]

Supplement Figure 4

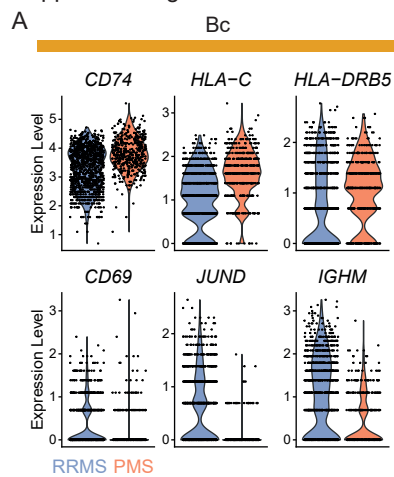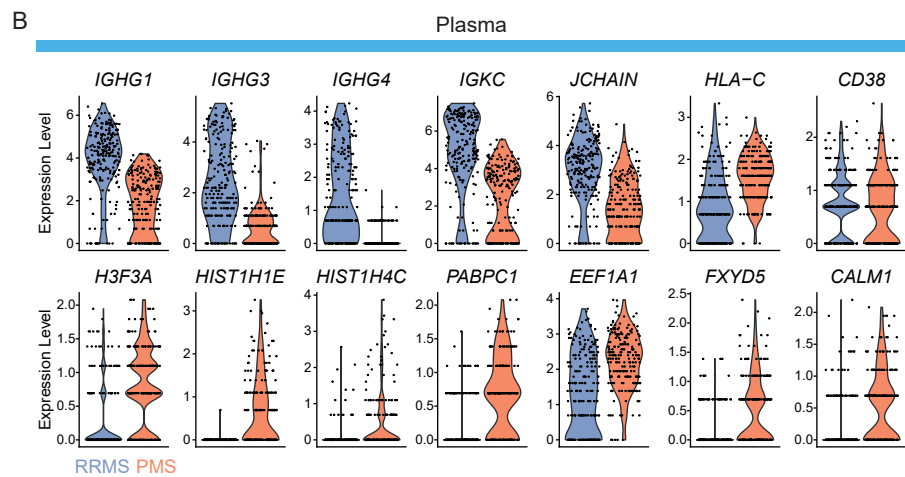

Supplement: Supplementary file 7 — Supplementary Material 7. [file 12974_2026_3861_MOESM7_ESM.pdf]
